# A Case Report of Pseudomyxoma Peritonei Arising From Primary Mucinous Ovarian Neoplasms

**DOI:** 10.7759/cureus.29309

**Published:** 2022-09-19

**Authors:** Reuben Joseph, Ramprasath Sathiamoorthy, Rajkumar Gnanasekaran, Lavanya Gunasekaran, Gurubharath Ilangovan

**Affiliations:** 1 Radiology, Chettinad Hospital and Research Institute Chettinad Academy of Research and Education, Chennai, IND

**Keywords:** academic radiology, abdominal radiology, pseudomyxoma peritonei, ovary, mucinous neoplasms, cystic mass, appendix

## Abstract

Pseudomyxoma peritonei (PMP) is a rare manifestation of primary mucinous neoplasms. We report two rare cases of PMP originating from mucinous primary ovarian neoplasms. The case series discusses the cases of female patients aged 86 and 52 years who presented with worsening dyspepsia, abdominal distension, pelvic pain, and altered bowel habits. Both of the patients underwent evaluation comprising cancer antigen-125 (CA-125) levels, ultrasound (US) examination of the abdomen and the pelvis, tumor markers, cytological evaluation, and contrast-enhanced computed tomography (CECT) of the pelvis and abdomen. Patients were diagnosed to have pseudomyxoma peritonei arising from mucinous ovarian tumors. Patients were referred to the surgical department and were successfully managed with repeated removal of mucinous material. The present case report highlights the significant radio-pathologic characteristics of PMP, which originated from mucinous ovarian tumors.

## Introduction

Pseudomyxoma peritonei (PMP) is a rare disease arising from primary mucinous tumors, which are usually appendiceal mucinous epithelial neoplasms and rarely mucinous ovarian tumors [1]. PMP is exceedingly rare, with a reported incidence of one to two individuals per million people per year [2]. Common presenting symptoms of PMP are abdominal pain associated with pelvic or abdominal masses and distension of the abdomen. The current case report discusses the radio-pathologic features of two post-menopausal patients aged 86 and 52 years who presented with major complaints of progressively worsening indigestion and abdominal distension for two to three months.

## Case presentation

Case report 1

**Figure 1:**
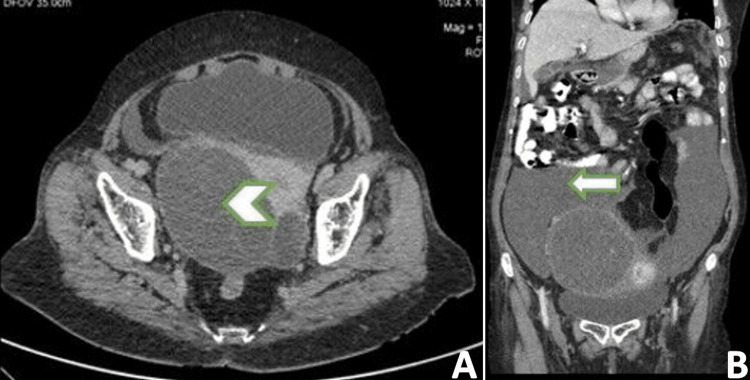
Contrast-enhanced computed tomography of abdomen and pelvis: (A) axial view and (B) coronal view CECT of abdomen and pelvis showed (A) a large peripherally enhancing cystic mass arising from the right ovary with thin enhancing septae (arrowhead); (B) mucinous fluid within the peritoneal cavity (white arrow).

An 86-year-old female patient presented with progressively worsening indigestion and abdominal distension for two to three months and was referred to the department of radiology for the evaluation of a pelvic mass. Ultrasound examination of the abdomen and pelvis revealed a multiseptated and large cystic mass in the right ovary with severe ascites in the abdomen. The expression level of the tumor marker cancer antigen-125 (CA-125) was 61.2 U/ml (normal range: 0-35 units/mL). Contrast-enhanced computed tomography (CECT) of the abdomen and pelvis showed a large, peripherally enhancing cystic mass in the right ovary with thin enhancing septae. A similar cyst was noted, inseparable from the left ovary (Figure 1).

**Figure 2:**
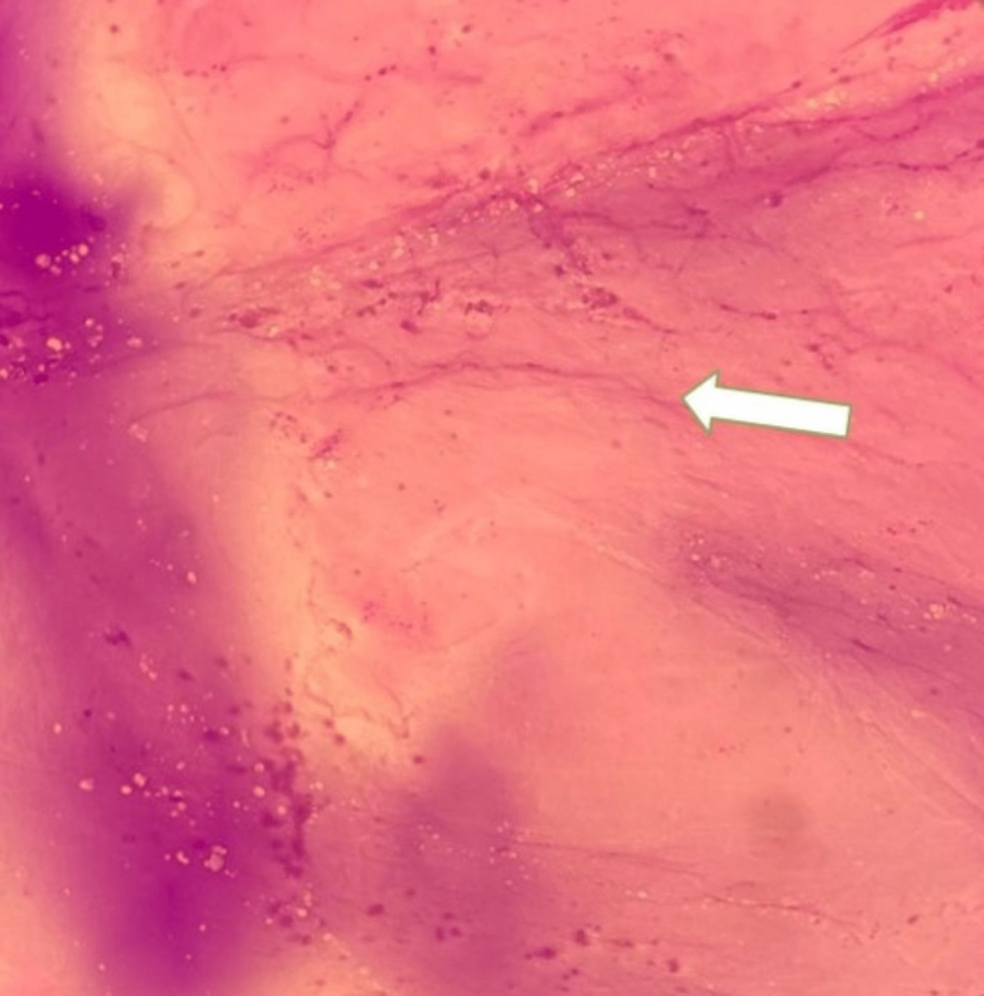
Aspirated ascitic fluid smear Aspirated ascitic fluid showing mucinous epithelium totally lacking cytologic atypia consistent with pseudomyxoma peritonei (white arrow).

There were massive loculated ascites involving the inframesocolic space and paracolic gutter (bilaterally) with nodular peritoneal thickening and fat stranding. Around 20 ml of ascitic fluid was aspirated for further histopathological evaluation, which revealed mucinous epithelium totally lacking cytologic atypia consistent with PMP (Figure 2).

**Figure 3:**
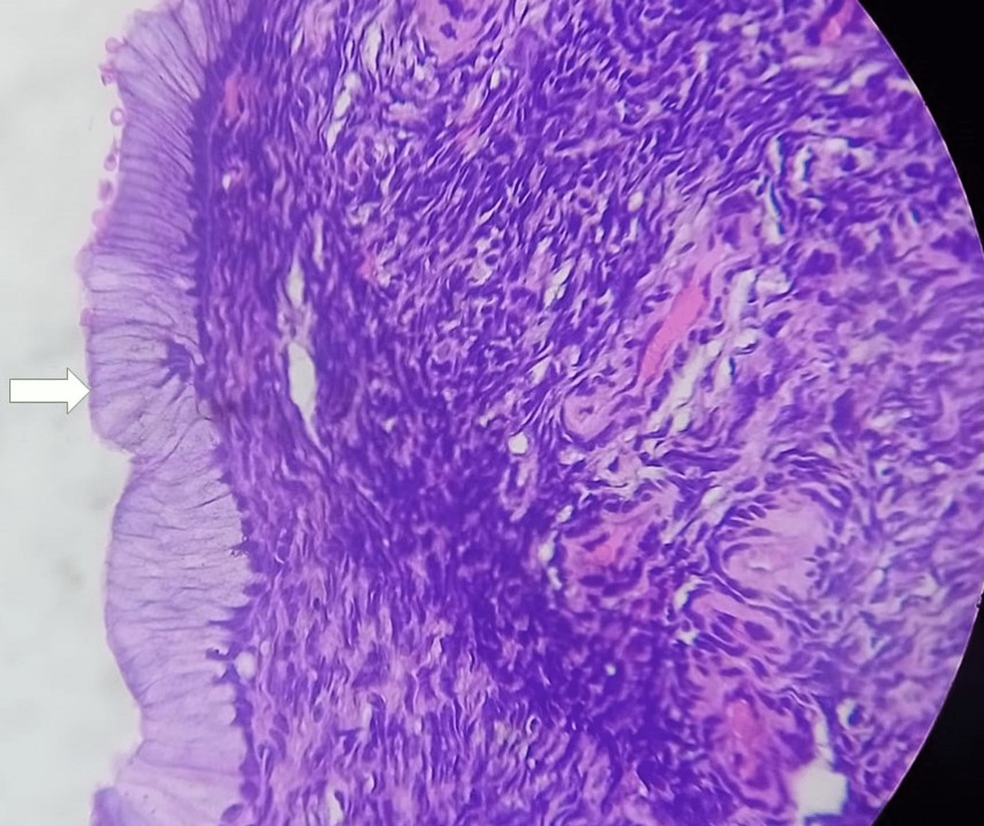
Mucinous ovarian cystadenoma Ovarian lesion showing simple, non-stratified, mucinous epithelium with small basally located nuclei lacking cytological atypia consistent with benign mucinous ovarian cystadenoma (white arrow).

The ovarian lesion was sent for further histopathological evaluation, which showed simple, non-stratified, mucinous epithelium with small basally located nuclei lacking cytological atypia, consistent with benign mucinous ovarian cystadenoma (Figure 3).

The patient was referred to the surgical department for further management.

Case report 2

**Figure 4:**
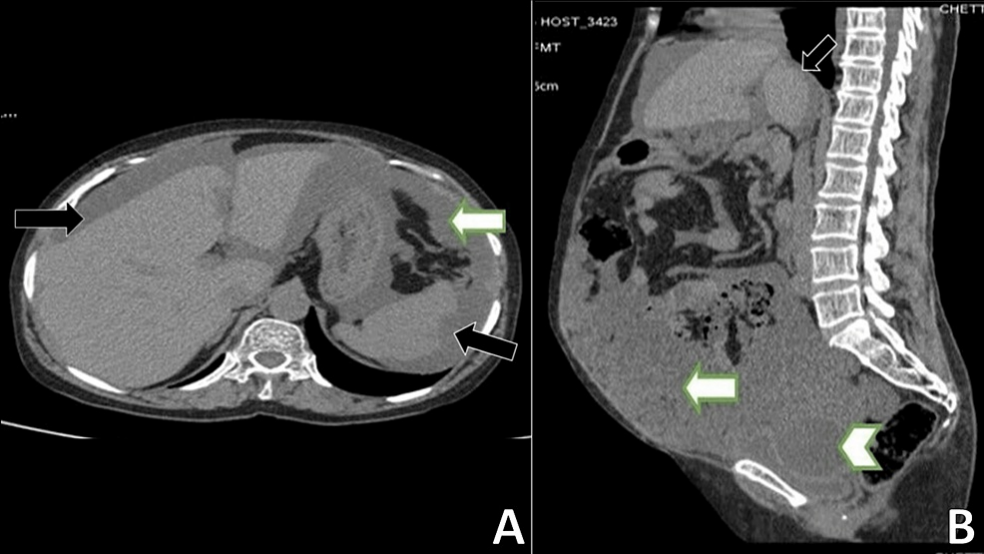
CT abdomen and pelvis: (A) axial view and (B) sagittal view Mucinous appearing free fluid in peritoneal cavity (white arrow) and scalloping of liver and spleen (black arrow) (A). CT abdomen and pelvis showed (B) a complex cystic mass with multiple septations in the pelvis posterior to the bladder causing indentations on the bladder contour (arrowhead). There was mucinous appearing free fluid in the peritoneal cavity (white arrow).

A 52-year-old female patient presented with similar complaints of abdominal distension, progressively worsening indigestion, pelvic pain, and a change in bowel habits over a period of three months. The pain was non-radiating, dull aching, and was not relieved with medications. There was no history of fever either. A CT scan of the abdomen and pelvis was performed, which showed a complex cyst with multiple septations in the pelvis located posterior to the bladder, causing indentations on the bladder contour (Figure 4).

**Figure 5:**
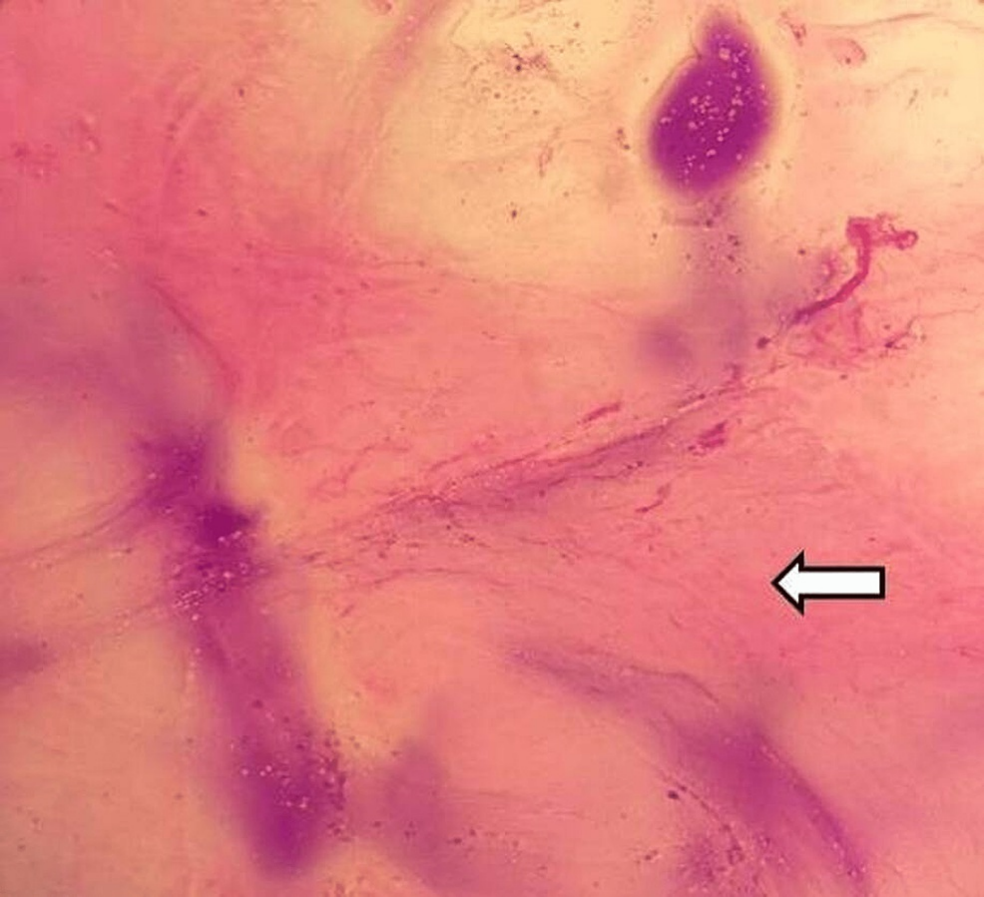
Aspirated ascitic fluid Ascitic fluid aspirated showed acellular pink proteinaceous mucus material with few RBCs in the background consistent with pseudomyxoma peritonei (white arrow). RBC: red blood cells.

The mass was partially encasing segments of the small bowel in the vicinity. The uterus and ovaries could not be imaged separately. There was extensive greater omental thickening (omental caking). Nodular peritoneal thickening was also noted, especially along the bilateral paracolic gutter. The surface of the liver and spleen adjacent to the diaphragm showed scalloping, which was suggestive of visceral peritoneal seedings. Scalloping of the liver surface was noted along the falciform ligament as well. Nodular deposits were noted on the surface of small bowel loops, especially in the right iliac fossa. There was moderate ascites entrapping bowel loops in the middle. Fluid aspiration was done, which showed acellular pink proteinaceous mucus material with a few RBCs in the background, consistent with pseudomyxoma peritonei (Figure 5).

**Figure 6:**
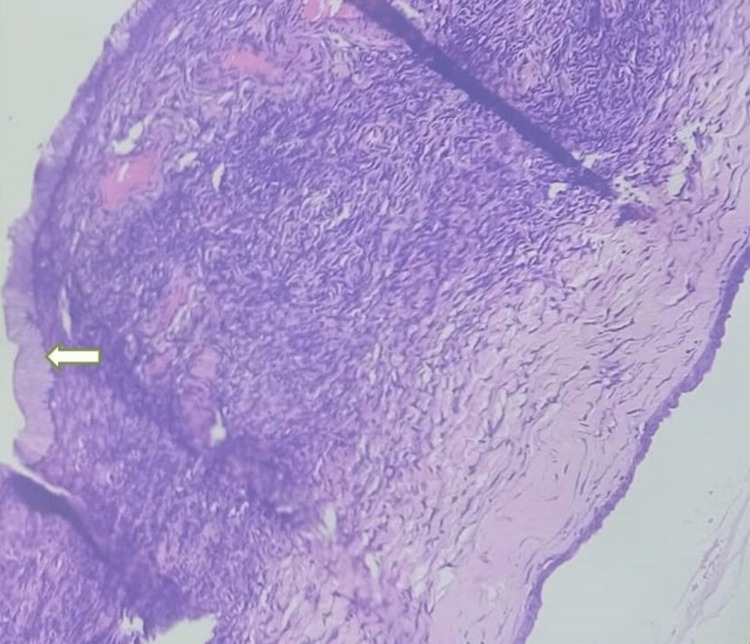
Benign mucinous ovarian cystadenoma Ovarian lesion showing a cyst wall lined by single layer of mucinous epithelium (white arrow) consistent with benign mucinous ovarian cystadenoma.

Further, a histopathological examination of the pelvic mass was done, which showed an ovarian lesion composed of thin-walled cysts with mucinous columnar cells having basally oriented nuclei, consistent with benign mucinous ovarian cystadenoma (Figure 6).

The patient was referred to the surgery department for further management.

## Discussion

PMP appears to be more common in women as a result of the trans-coelomic spread of ovarian tumors. Female patients also tend to present early due to the aggressive enlargement pattern of ovarian mucinous tumors giving rise to early symptoms such as abdominal pain and swelling [2]. Due to the suspicion of ovarian tumors in women, there is extensive use of CT scan in women and hence women present early. On the other hand, males are usually symptom-free initially, hence often present at an advanced stage [2]. 

The self-contained nature of the tumor rupture which limits microbial contamination by closing communications to the gut is the likely cause of the disease's asymptomatic nature. However; abdominal distension, discomfort, and pain are the late-onset symptoms brought on by the slow-moving fluid build-up caused by omental caking and pelvic masses. Such disease progression leads to malnutrition among patients. Also, bowel obstruction and compromise of the respiratory functions occur setting off a cascade of terminal events [3].

PMP appears to be more common in women as a result of the trans-coelomic spread of ovarian tumors. Female patients also tend to present early due to the aggressive enlargement pattern of ovarian mucinous tumors, giving rise to early symptoms such as abdominal pain and swelling [2]. Due to the suspicion of ovarian tumors in women, there is extensive use of CT scans in women, and hence women present early. On the other hand, males are usually symptom-free initially, hence often present at an advanced stage [2].

The self-contained nature of the tumor rupture, which limits microbial contamination by closing communications to the gut, is the likely cause of the disease's asymptomatic nature. However, abdominal distension, discomfort, and pain are the late-onset symptoms brought on by the slow-moving fluid build-up caused by omental caking and pelvic masses. Such disease progression leads to malnutrition among patients. Also, bowel obstruction and compromise of respiratory functions occur, setting off a cascade of terminal events [3].

Several immunohistochemical markers such as CA-125, ER, PR, Pax8, and WT1 are known to be expressed at different levels in primary epithelial ovarian tumors [4]. Ca-125 was high in the 86-year-old patient. High CA-125 levels are associated with a poor prognosis for PMP patients. Fisken et al. also observed poor survival with escalating levels of CA-125 [5].

The CT scan reveals a mucinous ovarian tumor appearing as a large, unilateral, and complex cystic mass with variably attenuating fluid within the cyst loculi and internal calcifications. Features suggestive of an ovarian malignancy include a necrotic soft-tissue component with thick septa, thick irregular walls, and sometimes papillary projections. Other supportive findings include surrounding organ invasion, adenopathy, ascites, and involvement of the peritoneum, omentum, or mesentery. However, these findings are only specific to epithelial neoplasms and not to their subtype, which is mucinous neoplasms. In fact, most mucinous ovarian neoplasms are benign (4 in 5 cases) or borderline on pathologic analysis [6]. Ascites in a case of a mucinous tumor usually represents pseudomyxoma peritonei. In the instance where PMP and an ovarian lesion are found together, an appendiceal origin mucinous tumor must be searched for simultaneously.

CECT of the pelvis and abdomen is the gold-standard imaging of choice in the early staging of pre-treatment ovarian cancer and for detecting metastases to other organs. CT is supposed to be 94% accurate in the staging of ovarian cancer. However, limitations with CT include <5 mm metastases being undetectable when it involves the bowel surface, mesentery, and/or peritoneum. MRI continues to remain a problem-solving technique that is not used routinely and is not always necessary even though it has shown equal accuracy when compared to CT [7]. Diffusion-weighted sequences with low b‑values help visualize septa within ascitic fluid more clearly, which is an indicator for PMP [7]. Low b-values reduce the signal intensity of the high signal-intensity water molecules in ascitic fluid; hence the septa which show low signal intensity is better visualized.

It was estimated that the overall five-year survival rate for pseudomyxoma peritonei ranged between 11% and 75%. The median survival is 9.8 years and is independently associated with low-grade tumors [8,9]. In the study by Gough et al. [10], the findings revealed that among the prognostic factors, pseudomyxoma peritonei negatively affected survival.

## Conclusions

PMP is a rare clinical manifestation mostly associated with mucinous tumors. The case report presented is a rare clinical presentation arising from mucinous ovarian tumors, even though the most common cause is appendiceal tumors. These ovarian tumors are mostly benign or borderline, with a rare chance of malignancy. With the knowledge of classic imaging findings on CT, PMP can be diagnosed early on, which has been the aim of this case report. PMP has certain typical characteristics in CT that all radiologists should be aware of in order to increase the sensitivity of its detection. Confirmation of the origin of PMP is done through immunohistochemistry. Following this, the causative tumor is diagnosed histologically. FIGO system-staged mucinous ovarian neoplasms are then managed surgically (which includes cytoreductive surgery and, if needed, hyperthermic intraperitoneal chemotherapy), thus giving a good long-term prognosis for patients.
